# Clinical Assessment of the Drug Interaction Potential of the Psychotropic Natural Product Kratom

**DOI:** 10.1002/cpt.2891

**Published:** 2023-03-28

**Authors:** Rakshit S. Tanna, James T. Nguyen, Deena L. Hadi, Matthew E. Layton, John R. White, Nadja B. Cech, Nicholas H. Oberlies, Allan E. Rettie, Kenneth E. Thummel, Mary F. Paine

**Affiliations:** 1 Department of Pharmaceutical Sciences, College of Pharmacy and Pharmaceutical Sciences, Washington State University, Spokane, Washington, USA; 2 Center of Excellence for Natural Product Drug Interaction Research, Spokane, Washington, USA; 3 Elson S. Floyd College of Medicine, Washington State University, Spokane, Washington, USA; 4 Department of Pharmacotherapy, College of Pharmacy and Pharmaceutical Sciences, Washington State University, Spokane, Washington, USA; 5 Department of Chemistry and Biochemistry, University of North Carolina at Greensboro, Greensboro, North Carolina, USA; 6 Department of Medicinal Chemistry, School of Pharmacy, University of Washington, Seattle, Washington, USA; 7 Department of Pharmaceutics, School of Pharmacy, University of Washington, Seattle, Washington, USA.

## Abstract

Oral formulations prepared from the leaves of the kratom (*Mitragyna speciosa*) plant are increasingly used for their opioid-like effects to self-manage opioid withdrawal and pain. Calls to US poison centers involving kratom exposures increased >50-fold from 2011–2017, one-third of which reported concomitant use of kratom with drugs of abuse. Many of these drugs are eliminated primarily *via* cytochrome P450 (CYP) 3A and CYP2D6, raising concerns for potential adverse pharmacokinetic kratom-drug interactions. The impact of a single low dose of kratom tea (2 g) on the pharmacokinetics of the CYP3A probe midazolam (2.5 mg) and CYP2D6 probe dextromethorphan (30 mg) were assessed in 12 healthy adult participants after oral administration. Kratom showed no effect on dextromethorphan area under the plasma concentration time-curve (AUC) and maximum concentration (*C*_max_; geometric mean ratio (90% confidence interval) 0.99 (0.83–1.19) and 0.96 (0.78–1.19), respectively) but a modest increase in midazolam AUC and *C*_max_ (1.39 (1.23–1.57) and 1.50 (1.32–1.70), respectively). Lack of change in midazolam half-life (1.07 (0.98–1.17)) suggested that kratom primarily inhibited intestinal CYP3A. This inference was further supported by a physiologically based pharmacokinetic drug interaction model using the abundant alkaloid mitragynine, a relatively potent CYP3A time-dependent inhibitor *in vitro* (*K*_*I*_, ~4 μM; *k*_*inact*_, ~0.07 min^−1^). This work is the first to clinically evaluate the pharmacokinetic drug interaction potential of kratom. Co-consuming kratom with certain drugs extensively metabolized by CYP3A may precipitate serious interactions. These data fill critical knowledge gaps about the safe use of this increasingly popular natural product, thereby addressing ongoing public health concerns.

Kratom (*Mitragyna speciosa* (Korth.) Havil.), a medicinal plant belonging to the coffee family (Rubiaceae), traditionally has been used by manual laborers in Southeast Asia to self-treat fatigue and pain.^1^ Dried kratom leaf powder is commonly consumed as a tea or capsule or is swallowed with a drink. Consumption of kratom leaf preparations produces dose-dependent pharmacological effects. At lower doses (1–5 g), kratom produces a mild stimulating effect analogous to caffeine, whereas, at moderately higher doses (5–15 g), opioid-like effects can ensue. Based on its psychoactive effects, kratom is increasingly used as a natural and presumably safe alternative for managing pain and opioid withdrawal symptoms.^2^

Alkaloids present in kratom leaves are believed to elicit psychoactive effects following ingestion. Mitragynine is typically the most abundant alkaloid in kratom leaves^3^ and derived products. Other key kratom alkaloids include its diastereoisomers speciogynine, speciociliatine, and mitraciliatine and the structurally related analogues paynantheine and isopaynantheine.^3–5^ These alkaloids, along with their metabolites, including 7-hydroxymitragynine, differentially engage with opioid (*μ*, *κ*, and *δ*), adrenergic (*α*_1_ and *α*_2_), and serotonergic (5-HT_1A_ and 5-HT_2B_) receptors, resulting in an “entourage effect.”^4,6–9^

The widespread availability of kratom *via* internet vendors or at gas stations, head shops, smoke shops, and kratom-specific cafés has led to a marked increase in usage over the last decade.^10^ This surge in use led to a more than 50-fold increase in the annual number of calls to US poison centers involving kratom exposures from 2011–2017 (13 to 682), one-third of which reported use with drugs of abuse. Most of these drugs were opioids and benzodiazepines, several of which are extensively metabolized by cytochrome P450 (CYP) 2D6 and CYP3A.^11^ Blood from autopsies of 14 of 15 patients due to overdose deaths in Colorado associated with kratom exposure tested positive for these and other drugs.^12^ According to a Centers for Disease Control and Prevention report, 152 overdose deaths from July 2016 to December 2017 tested positive for mitragynine. However, only seven decedents tested negative for the presence of concomitant drugs upon postmortem toxicology reports.^13^

These deaths could have been caused by kratom or the concomitant drug(s), but the collective evidence suggests that these deaths might have involved kratom-drug interactions, which may be pharmacodynamic and/or pharmacokinetic in nature. To mitigate this safety concern, the US Drug Enforcement Administration (DEA) intended to classify mitragynine and 7-hydroxymitragynine as Schedule I controlled substances but later withdrew their intent due to public outcry about the purported medicinal benefits of kratom.^14^ The US Food and Drug Administration (FDA) subsequently called for more research to better understand the safety profile of kratom, especially in combination with other drugs.^15,16^

Kratom extracts, mitragynine, and other kratom alkaloids have been reported to inhibit several CYP enzymes *in vitro*, including CYP2D6 and CYP3A.^4,17,18^ Mitragynine showed reversible inhibition of hepatic CYP2D6 activity (*K*_*i*_, 1.17 ± 0.07 μM) and time-dependent inhibition of both hepatic (*K*_*I*_, 4.1 ± 0.9 μM; *k*_*inact*_, 0.068 ± 0.01 min^−1^) and intestinal (4.2 ± 2.5 μM; *k*_*inact*_, 0.079 ± 0.02 min^−1^) CYP3A activity in human liver and intestinal microsomes.^17^ Using the FDA-recommended mechanistic static static model, mitragynine was predicted to have a negligible effect on the ratio of the area under the plasma concentration vs. time curve of the object drug in the presence to absence of inhibitor (AUCR) of the CYP2D6 probe drug dextromethorphan (1.06) but a substantial effect on the AUCR of the CYP3A probe drug midazolam (5.69).^17^

The objective of this work was to clinically assess the pharmacokinetic drug interaction potential of kratom when co-consumed with pharmaceutical drugs extensively metabolized by CYP2D6 and CYP3A. The aims were to (i) evaluate the effects of a single low dose (2 g) of a well-characterized kratom product on the pharmacokinetics of dextromethorphan and midazolam in healthy adult participants and (ii) advance the mechanistic understanding of the resulting pharmacokinetic kratom-midazolam interaction using physiologically-based pharmacokinetic (PBPK) modeling and simulation. Results from this first-ever clinical kratom-drug interaction study provide direct evidence regarding the risk of co-consuming kratom with drugs extensively metabolized by CYP2D6 and CYP3A.

## MATERIALS AND METHODS

### Chemicals and reagents

Dextromethorphan, dextromethorphan-d_3_, dextrorphan, 1′-hydroxymidazolam, 4-hydroxymidazolam, 7-hydroxymitragynine, midazolam, midazolam-d_4_, mitragynine, and mitragynine-d_3_ were purchased from Cayman Chemical (Ann Arbor, MI). The kratom indole alkaloids speciociliatine, speciogynine, mitraciliatine, paynantheine, and isopaynantheine were isolated and purified from commercial kratom products as described^3,5^; all reference standards were verified to be ≥95% pure as measured by ultra-high-performance liquid chromatography-ultraviolet spectroscopy (UHPLC-UV) analysis. *tert*-Butyl methyl ether was purchased from Sigma-Aldrich (St. Louis, MO). Acetonitrile and methanol were purchased from Fisher Scientific (Fair Lawn, NJ). Blank human plasma (K_2_EDTA, pooled, mixed gender, lot HMN99236) was purchased from BioIVT (Westbury, NY). All other chemicals and reagents were analytical grade.

### Clinical pharmacokinetic kratom-drug interaction study

#### Kratom product selection and tea preparation.

A single large batch of the representative kratom product, coded K51 (described as Yellow Indonesian Micro Powder), was acquired and characterized as described.^19^ K51 contained 19.48 ± 0.81, 3.18 ± 0.13, 0.647 ± 0.035, 5.12 ± 0.26, 5.86 ± 0.26, 0.512 ± 0.010, and <0.124 ± 0.0014 (below the limit of quantification) mg of mitragynine, speciogynine, mitraciliatine, speciociliatine, paynantheine, isopaynantheine, and 7-hydroxymitragynine, respectively, in each g of dried leaf powder. K51 was prepared as a tea to emulate the most common method of kratom consumption. A single low dose of K51 (2 g) was added to a 350-mL Styrofoam cup, followed by 240 mL hot water (80°C), and the mixture was stirred. After the resulting tea was allowed to steep for 3 minutes, a sugar packet (4 g) was added to improve palatability. The prepared tea along with the residual powder was cooled to 50°C before administering it to the participants.

#### Clinical protocol and participants.

The Washington State University (WSU) Institutional Review Board approved the clinical protocol and informed consent form. The study was registered with the ClinicalTrials.gov database (NCT04392011) and was conducted at the WSU Clinical Research Unit on the Health Sciences Campus under Investigational New Drug status (#145002) and in accordance with the Code of Federal Regulations on the Protection of Human Subjects (45 CFR Part 46). Healthy adults previously exposed to kratom and willing to abstain from kratom use for several weeks were recruited. Potential participants provided written informed consent and Health Insurance Portability and Accountability Act authorization prior to screening. Eligibility to participate was assessed based on medical history, physical examination, routine clinical laboratory tests, urine pregnancy tests for females, and inclusion/exclusion criteria (Table S1). Drug toxicology screens for multiple common drugs of abuse, including several opioids and benzodiazepines, as well as urine pregnancy tests for females, were conducted the morning of each inpatient day prior to study procedures.

#### Study design.

**Figure 1:**
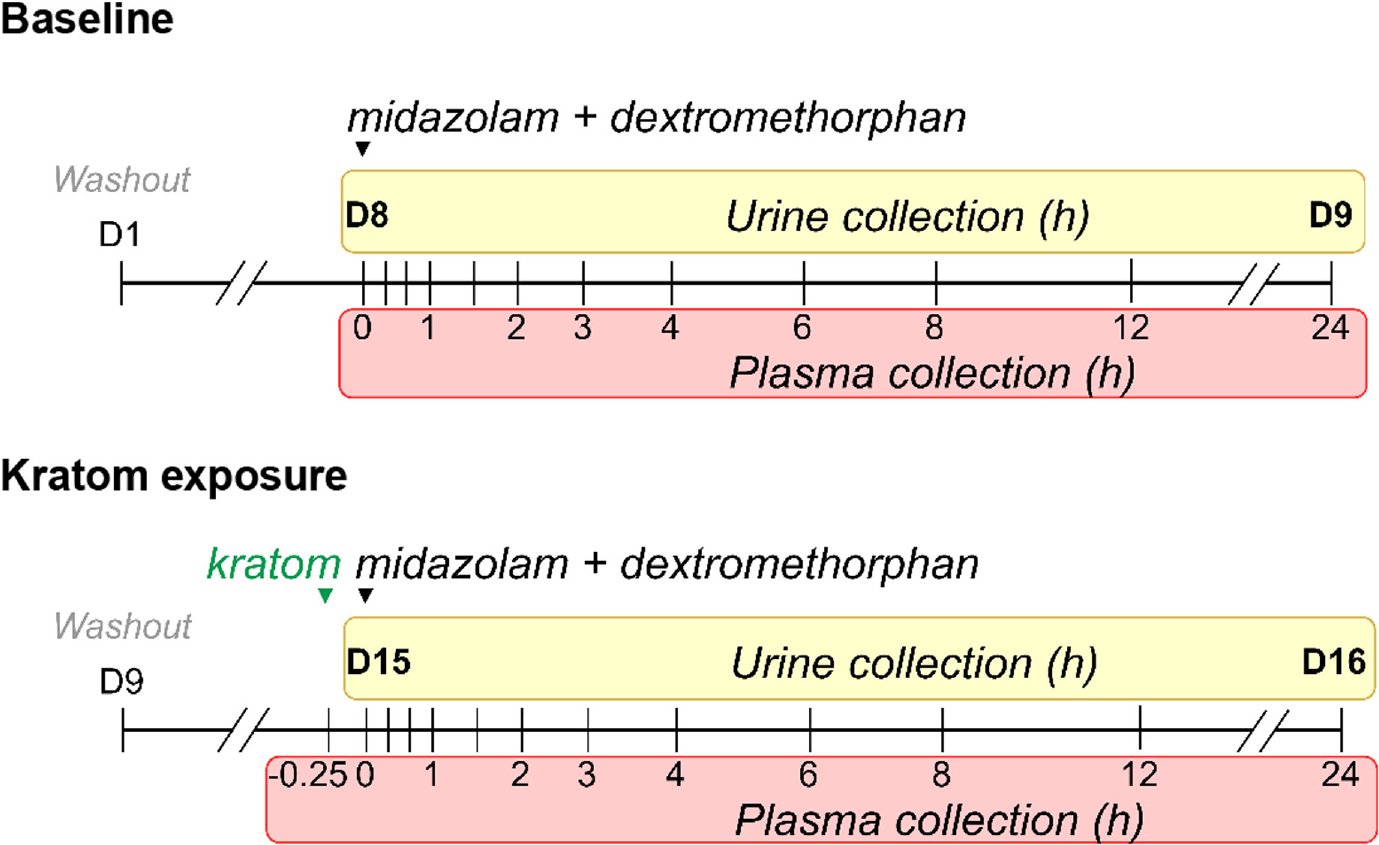
Clinical study design. Healthy adults (6 males, 6 females) participated in a two-arm open-label, fixed-sequence, crossover pharmacokinetic kratom-drug interaction study. During the baseline arm, after an overnight fast, participants were administered an oral cocktail consisting of midazolam (2.5 mg) and dextromethorphan (30 mg) as probes for CYP3A and CYP2D6 activity, respectively. During the kratom exposure arm, after an overnight fast, participants were instructed to drink 240 mL of kratom tea, prepared with a low dose (2 g) of Yellow Indonesian Micro Powder (K51), within 10 minutes. Participants were orally administered the same probe cocktail 15 minutes after kratom tea administration. Blood and urine were collected at the indicated times.

Healthy adult participants (6 males and 6 nonpregnant nonlactating females) were enrolled in an open-label, two-arm, fixed-sequence crossover study (Figure 1). During the baseline arm, following an overnight fast, they were orally administered 2.5 mg midazolam HCl (2 mg/mL syrup; West-Ward Pharmaceuticals, Eatontown, NJ) and 30 mg dextromethorphan HBr (22 mg of the free base, Robafen Cough Liquidgels; Rugby Laboratories, Livonia, MI) with 240 mL water. Blood (~5 mL) was collected in BD K_3_ EDTA-containing vacutainer tubes (Fisher Scientific, Pittsburgh, PA) through an indwelling venous catheter before and at 0.25, 0.5, 1, 1.5, 2, 3, 4, 6, 8, and 12 hours following drug administration. Participants continued to fast until after the 4-hour blood collection, when lunch was provided. They returned to the Clinical Research Unit 24 hours after drug administration for blood collection *via* venipuncture. Blood was immediately cooled on ice, and plasma was harvested by centrifugation (1,600 *g* × 10 minutes). Urine was collected from 0–12 hours. Upon discharge, participants were instructed to continue collecting their urine (12–24 hours) until returning for the 24-hour blood draw the next morning. Plasma and urine were stored at −80°C and −20°C, respectively, until bioanalysis.

During the kratom exposure arm, following an overnight fast, participants were administered kratom tea. They were instructed to drink the entire volume within <10 minutes, which included rinsing the cup with an additional 100 mL of water to ensure the consumption of residual tea powder. Fifteen minutes later, they were orally administered dextromethorphan and midazolam as described for the baseline arm. Blood was collected before kratom administration, at the time of probe drug administration, and from 0.25–24 hours following drug administration as described above. Urine was collected from 0–24 hours, and blood and urine were processed and stored as described above. Vital signs (blood pressure, heart rate, and oxygen saturation) were recorded before drug or tea administration, periodically throughout the inpatient days and during the outpatient visits. A washout period of at least 1 week separated the 2 arms. Epinephrine injection USP (0.3 mg; Teva Pharmaceuticals, North Wales, PA) and naloxone injection USP (1 mg/mL, International Medication Systems Ltd, South El Monte, CA) were available if needed to mitigate severe adverse events.

### Bioanalysis of plasma and urine samples

Plasma and urine samples were processed using liquid–liquid extraction as described.^19^ In brief, the samples were thawed on ice, briefly vortexed, and a 200-μL aliquot was transferred to a clean microcentrifuge tube. Plasma samples were mixed with 5 μL internal standard (dextromethorphan-d_3_, midazolam-d_4_, mitragynine-d_3_; 100 nM). Urine samples were mixed with 20 μL of 1 M acetate buffer (pH 5) and 5 μL internal standard before incubating with β-glucuronidase (2.5 μL 100,000 U/mL) for 20 hours. Plasma and urine samples were extracted with 600 μL *tert*-butyl methyl ether. After vortex-mixing and centrifugation, 500 μL supernatant was transferred to a clean microcentrifuge tube. The supernatant was evaporated to dryness and reconstituted with 50 μL methanol (50% v/v) and subjected to UHPLC–tandem mass spectrometry analysis. Analytes were quantified using a Shimadzu Nexera X2 UHPLC (Shimadzu Corporation, Tokyo, Japan) interfaced with the QTRAP 6500 system (AB Sciex, Framingham, MA) operating in positive electrospray ionization mode (Table S2).

### Pharmacokinetic analysis

The pharmacokinetics of the probe drugs and kratom alkaloids were determined using noncompartmental analysis methods within Phoenix WinNonlin (version 8.3; Certara, Princeton, NJ). The following were recovered from plasma concentration vs. time profiles as described^19^: maximum plasma concentration (*C*_max_), time to reach *C*_max_ (*t*_max_), terminal elimination rate constant (*λ*_*z*_), terminal elimination half-life (*t*_1/2_), area under the plasma concentration-time curve (AUC) from time zero to the time of the last measurable concentration (AUC_last_), AUC from time zero to infinity (AUC_inf_), oral clearance (CL/F), and apparent volume of distribution during the terminal phase (V_z_/F). Samples at the later timepoints that were below the lower limit of quantitation were treated as missing to avoid inappropriate estimation of *λ*_*z*_. Metabolite-to-parent AUC_inf_ (AUC_inf,m_/AUC_inf,p_) ratios for midazolam and dextromethorphan were calculated to compare the relative abundance of metabolite to parent drug in the circulation. The following were recovered from urinary concentrations of the probe drugs/metabolites as described^19^: cumulative amount excreted into urine during each collection interval (*A*_e,interval_), total amount excreted into the urine (*A*_e,total_), fraction of the administered dose excreted unchanged in the urine (*f*_*e*_), and renal clearance (CL_R_). Pharmacokinetic data are reported as geometric means (90% confidence intervals) or median (range) as appropriate.

### Statistical analyses

A sample size of 12 participants provided 80% power to detect a 25% change in the primary end point with a type I error of 0.05, assuming a 25% intra-individual variability in midazolam AUC.^20^ The primary end point was the geometric mean ratio of midazolam AUC_inf_ in the presence to absence of kratom tea. A pharmacokinetic interaction was considered evident if the geometric mean ratio of the primary end point lay outside the predefined no-effect range (0.75–1.33). Secondary end points included the AUC_inf_ for dextromethorphan and *C*_max_, *t*_1/2_, *t*_max_, and CL/*F* for dextromethorphan, midazolam, and their respective metabolites. Statistical comparisons were made using a paired Wilcoxon signed rank test. *P* values <0.05 were considered significant.

### Physiologically based pharmacokinetic modeling and simulation

PBPK models were developed for midazolam, dextromethorphan, and mitragynine using the population-based simulator Simcyp (version 22; Simcyp, Sheffield, UK). Mitragynine was used as a surrogate for kratom due to being the most abundant alkaloid in the K51 product,^5^ as well as a potent reversible inhibitor of CYP2D6 activity and time-dependent inhibitor of CYP3A activity in intestinal and/or liver microsomes.^17^ Mitragynine PBPK model development and input parameters are detailed in Table S3. The midazolam and dextromethorphan models within the Simcyp library were modified as described (Table S3).^21,22^ CYP2D6 phenotype of the healthy volunteer (sim-healthy volunteers) population was modified to match the phenotype of the participants from the clinical study.

The midazolam, dextromethorphan, and mitragynine PBPK models were verified using data from the current pharmacokinetic kratom-drug interaction study. The mitragynine model was next coupled with the midazolam and dextromethorphan models using the respective inhibition mechanisms and kinetic parameters recovered previously.^17^ Simulated pharmacokinetic data are reported as geometric means (90% confidence intervals) or median (range) as appropriate. Goodness-of-fit was evaluated by visual inspection of simulated and observed concentration-time profiles. Model performance was considered successful if the simulated pharmacokinetic endpoints (AUCR, AUC, and *C*_max_) were within 0.5 to 2-fold of the observed end points.

## RESULTS

## Participants and safety and tolerability of probe drugs and kratom tea

Of the 21 potential participants screened, 15 were enrolled. One female participant was withdrawn due to vomiting after consuming kratom tea, and the other two were withdrawn due to scheduling conflicts. The 12 participants who completed the study self-identified as White/non-Hispanic (3 males and 5 females), Black/non-Hispanic (1 male), Asian/non-Hispanic (1 male and 1 female), or multiracial/non-Hispanic (1 male). Median (range) participant age and weight were 30 (21–48) years and 76 (52–119) kg, respectively. None of the participants failed drug toxicology screening, and all abstained from taking concomitant medications and botanical or other natural products during the entire study period. The kratom tea and probe drugs were well tolerated by all 12 participants, and none experienced any severe adverse events. Five participants experienced headache or lightheadedness during at least one arm that did not result in study discontinuation. Relative to the baseline arm, kratom exposure had no effect on vital signs of the 12 participants (heart rate, blood oxygen saturation, and blood pressure; Figure S1).

### Pharmacokinetics of probe drugs and kratom alkaloids

**Figure 2:**
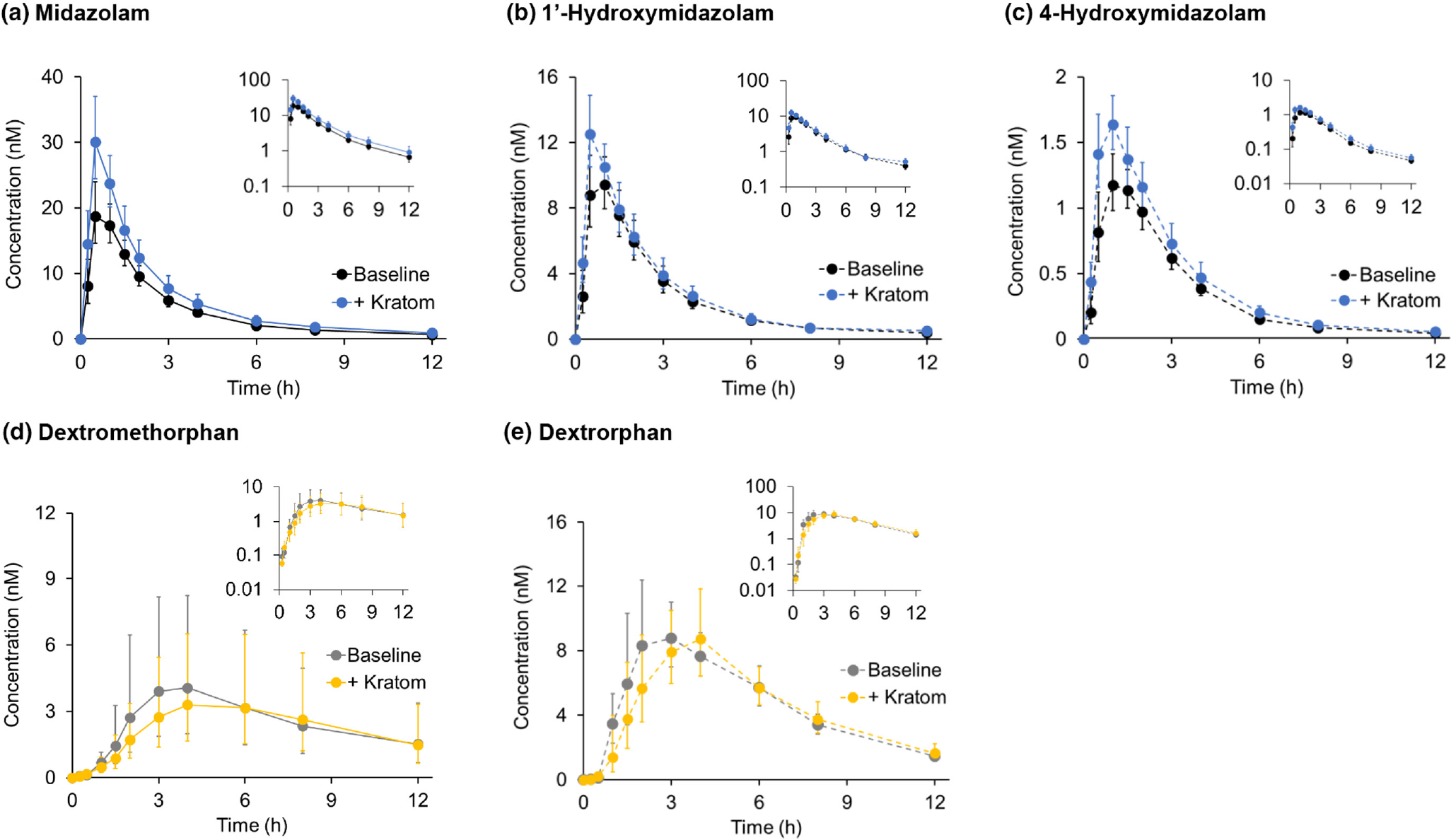
Plasma concentration-time profile for (**a**) midazolam, (**b**) 1′-hydroxymidazolam, (**c**) 4-hydroxymidazolam, (**d**) dextromethorphan, and (**e**) dextrorphan following a single oral dose of midazolam (2.5 mg) and dextromethorphan (30 mg) administered alone (black and gray symbols) or 15 minutes after kratom tea (2 g) administration (blue and yellow symbols). Symbols and error bars denote geometric means and 90% confidence intervals, respectively.

**Figure 3:**
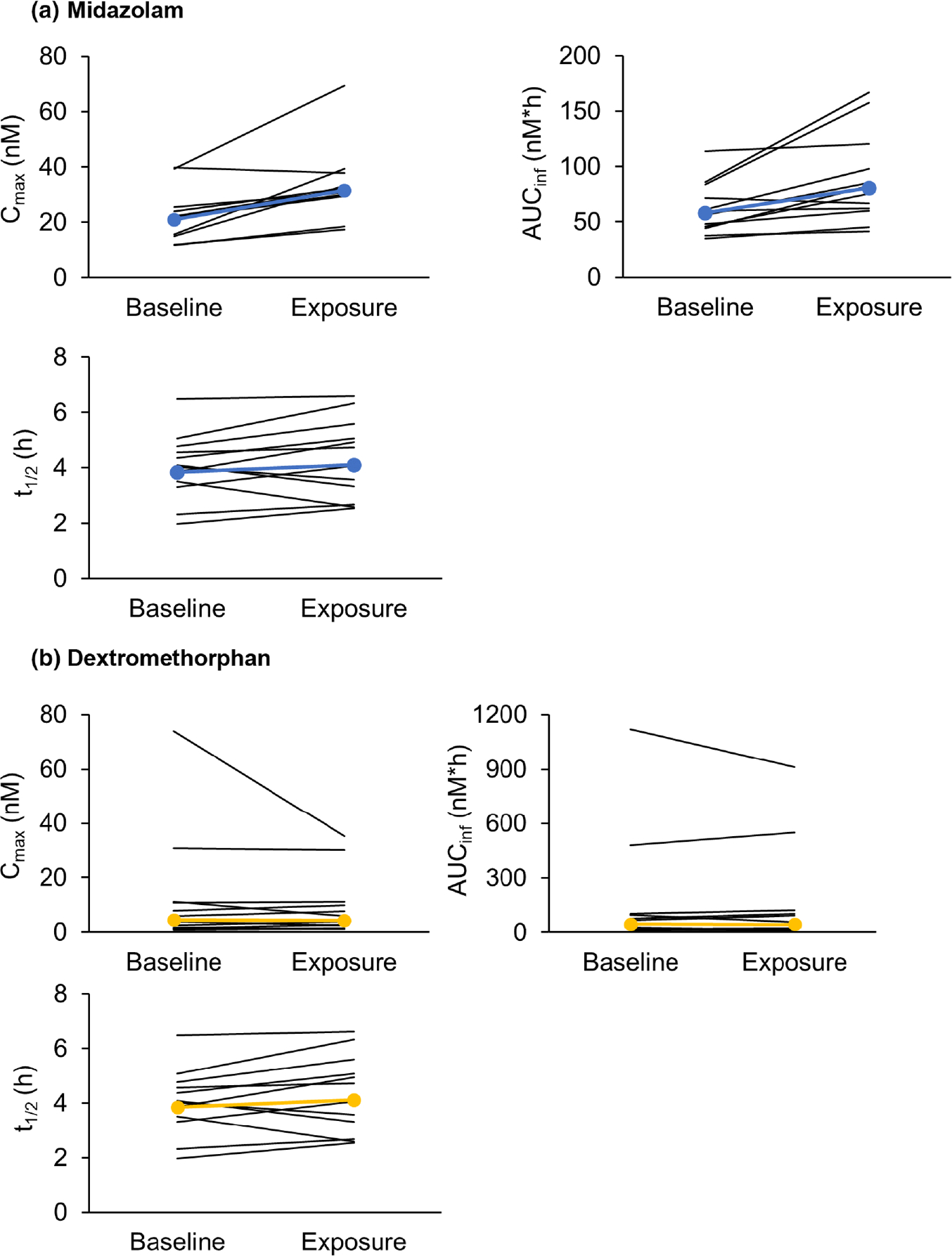
Effects of a single low dose (2 g) of kratom tea on the *C*_max_, AUC_inf_, and *t*_1/2_ of (**a**) midazolam and (**b**) dextromethorphan in 12 healthy adult participants following oral administration of midazolam (2.5 mg) and dextromethorphan (30 mg). Solid black lines denote individual values and blue and yellow symbols connected by lines denote geometric means. AUC_inf_, area under the plasma concentration-time profile from time zero to infinity; *C*_max_, maximum concentration; *t*_½_, terminal elimination half-life.

Relative to baseline, the kratom tea increased midazolam geometric mean AUC_0–12_, AUC_inf_, and *C*_max_ by 40–50% and had no effect on *t*_1/2_ and median *t*_max_ (Figure 2, Table 1). The geometric mean ratio for the primary end point, midazolam AUC_inf_ in the presence to absence of kratom, lay outside the predefined no-effect range (0.75–1.33). Kratom increased midazolam AUC_inf_ and *C*_max_ in all but one participant (Figure 3). Kratom increased the AUC_inf_ of the primary CYP3A-mediated metabolites, 1′-hydroxymidazolam and 4-hydroxymidazolam, by ~18–27% and decreased the metabolic ratios by ~10–15% (Figure 2, Table 1). Kratom had no effect on dextromethorphan and dextrorphan pharmacokinetics and the metabolic ratio. Due to the low renal excretion and clearance of midazolam and dextromethorphan, changes in urine pharmacokinetics were considered inconsequential. The AUC_0–24h_, *C*_max_, and *t*_max_ for mitragynine and other kratom alkaloids (Table S4) were consistent with our previous clinical kratom pharmacokinetic study.^19^ The *t*_1/2_ could not be recovered for any of the alkaloids due to blood collections being limited to 24 hours (i.e., < 3 half-lives).

### Physiologically based pharmacokinetic modeling and simulation

**Figure 4:**
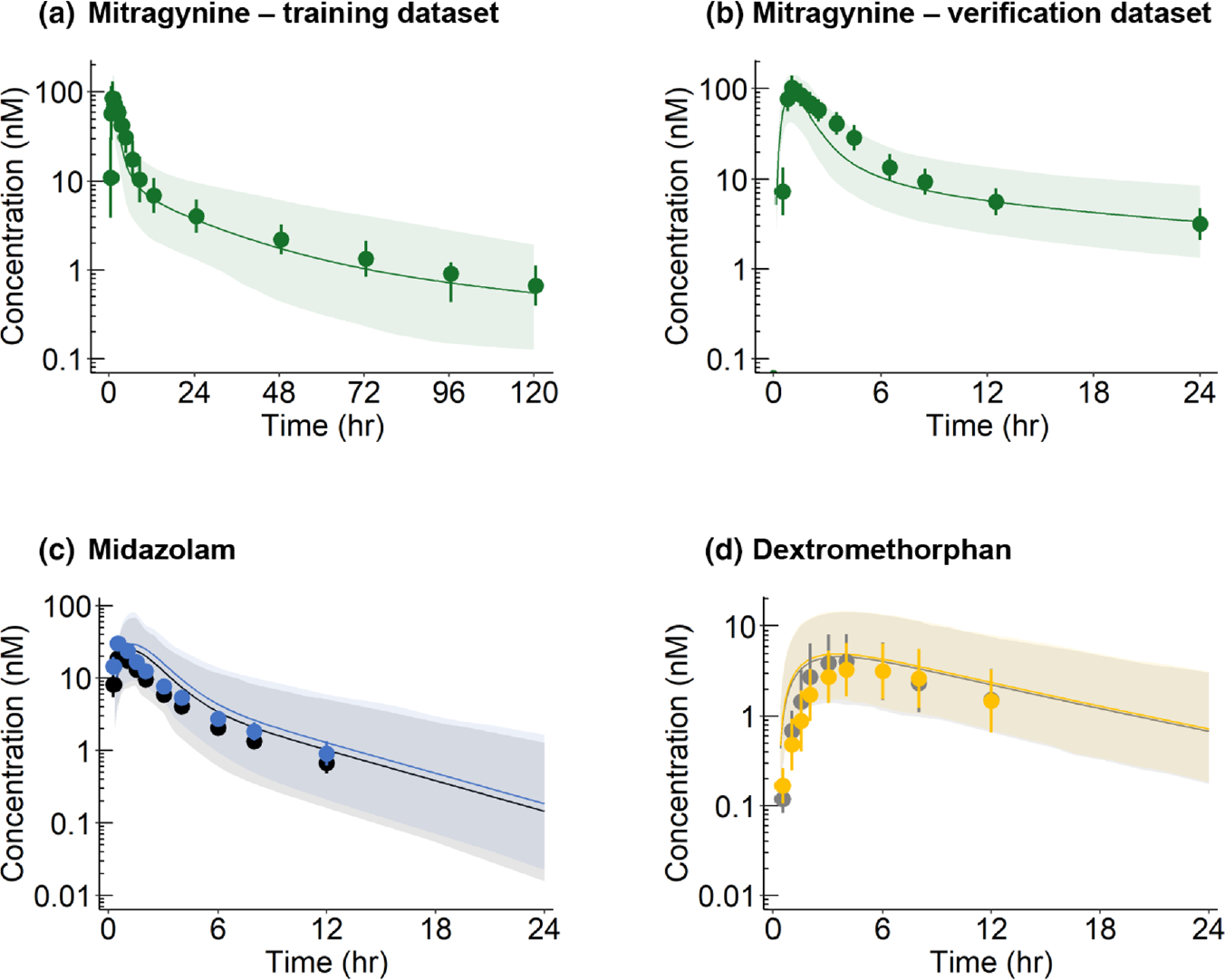
Simulated and observed concentration-time profiles of mitragynine (**a**) training and (**b**) verification datasets. Overlay of simulated and observed concentration-time profiles of (**c**) midazolam and (**d**) dextromethorphan before (black and gray) and after (blue and yellow) mitragynine (surrogate for kratom) administration where inhibition of CYP3A and CYP2D6 were incorporated. Symbols and error bars denote geometric means and 90% confidence intervals, respectively, of the observed data. Lines denote the geometric means, and the shaded regions represent the 5^th^ and 95^th^ percentiles of the simulated profile.

Ten trials of 10 virtual healthy participants (5 males) were simulated. The healthy participant population within the Simcyp simulator was modified to include ~17% intermediate CYP2D6 metabolizers and ~83% extensive CYP2D6 metabolizers based on the urinary log (dextromethorphan/dextrorphan) ratios of the clinical study participants (data not shown). The observed midazolam and dextromethorphan concentration-time profiles were captured within the 5^th^ and 95^th^ percentiles of the simulated profiles of the probe drugs administered alone (Figure 4). The mitragynine model, optimized using a logP of 4.8, captured the observed biphasic profile up to 120 hours from our previous (training dataset) study. The observed mitragynine concentration-time profile from this study (verification dataset) was also captured within the 5^th^ and 95^th^ percentiles of the simulated profiles (Figure 4). The model-derived pharmacokinetic outcomes (AUC and *C*_max_) were within 50% of the observed values (Table 2).

Simulation of the kratom-midazolam interaction using mitragynine as a surrogate for kratom showed an ~1.25-fold increase in AUC and ~1.2-fold increase in *C*_max_ for midazolam (Figure 4), which were ~15–20% lower than the observed values. The model successfully replicated the lack of change in midazolam *t*_1/2_. Simulated time-dependent inhibition of CYP3A by mitragynine led to a decrease in active CYP3A enzyme in the small intestine but not in the liver (Figure S2). Simulation of the kratom-dextromethorphan interaction successfully replicated a lack of interaction (Figure 4). The model-derived AUCR for both probe drugs was within ~10% of the observed AUCR.

## DISCUSSION

An estimated 2.1 million US residents in 2020 used kratom to self-manage pain and/or opioid withdrawal symptoms.^23^ Compared with nonusers or users of cannabis, alcohol, or cigarettes, kratom users have been reported to have a serious substance abuse profile that includes concomitant use of licit or illicit drugs, such as opioids and benzodiazepines.^24^ Polysubstance use was observed in 87% of the 156 kratom-related overdose fatalities,^25^ suggesting concerns for pharmacokinetic and/or pharmacodynamic adverse kratom-drug interactions.

Kratom has been shown to inhibit CYP enzymes *in vitro*, including CYP2D6 and CYP3A, which extensively metabolize several opioids and benzodiazepines.^17,18^ An *in vitro-in vivo* extrapolation based on a mechanistic static model suggested that mitragynine (equivalent to a single 2 g kratom dose) would precipitate a 5.7-fold increase in midazolam AUC.^17^ Although static models are known to overpredict the magnitude of an interaction, they provide decision-making information for further investigation.^26^ In addition, no human study has been conducted to evaluate the drug interaction potential of kratom. Collectively, the purpose of this work was to address this knowledge gap *via* a clinical pharmacokinetic kratom-drug interaction study involving healthy adult participants.

Midazolam and dextromethorphan were used as sensitive *in vivo* probe drugs for CYP3A and CYP2D6 activity, respectively. Simultaneous oral administration of midazolam (2 mg) and dextromethorphan (30 mg) were shown previously not to cross-interact and thus were administered as a cocktail.^27^ The probe drugs were coupled with a well-characterized kratom product representative of several other marketed kratom products.^4^ The selected dose (2 g) was within the low end of typical doses (1–3 g) used for the psychoactive effects of kratom.^28^

The kratom product, prepared as tea, modestly increased midazolam AUC_inf_ (~40%) and *C*_max_ (50%) and decreased CL/F (~25%) and V_z_/F (~25%; Figure 2, Table 1). Systemic exposure to the CYP3A-mediated metabolites 1′-hydroxymidazolam and 4-hydroxymidazolam also increased (AUC_inf_, ~18–27%) but by a smaller magnitude compared with midazolam. Although this increase in systemic exposure to the metabolites seems counterintuitive with respect to CYP3A inhibition, the inhibition of their secondary metabolism *via* UGT2B4/2B7 by kratom is a potential explanation. A kratom extract and mitragynine were previously shown to inhibit UGT2B7 (half-maximal inhibitory concentration, 8.11 μM) activity in human liver microsomes.^29^ Nonetheless, the ~10–15% decrease in 1′-hydroxymidazolam/midazolam and 4-hydroxymidazolam/midazolam AUCR in the presence of kratom, further indicated reduced CYP3A activity (Table 1). The lack of change in midazolam *t*_1/2_ suggested inhibition of first-pass metabolism, primarily CYP3A in the intestine (Figure 2). The higher increase in *C*_max_ compared with AUC_inf_ further supported the likelihood that at the given dose, kratom primarily impaired intestinal CYP3A activity akin to the grapefruit juice-felodipine interaction among other interactions.^30^

In contrast to the effects of the single low dose of kratom tea on midazolam systemic exposure, there were no effects on dextromethorphan exposure. Exposure to the CYP2D6-mediated primary metabolite, dextrorphan, also remained unaffected (Figure 2, Table 1). A noticeably slowed absorption of both dextromethorphan and dextrorphan was observed (Figure 2), which may have been due to a prolonged intestinal transit time elicited by kratom. Prolongation of intestinal transit time was also speculated in our previous clinical study for the gradations observed in the early (absorption) phase of the plasma concentration-time profiles of kratom alkaloids.^19^ This effect may occur *via* the interaction between kratom and intestinal opioid receptors.^31^ Further investigation is needed to address this potential mechanism.

The lack of effect of kratom tea on CYP2D6 activity despite potent inhibition *in vitro* may have reflected insufficient alkaloid concentrations at the enzymatic site in the liver. Alkaloid plasma concentrations were quantified, indicating the alkaloids are bioavailable (Figure S3, Table S4). In addition, the corresponding pharmacokinetics were consistent with those observed from our previous study^19^ when kratom was administered alone, suggesting that neither probe drug altered alkaloid disposition.

The current work is the first to use a PBPK modeling approach to gain mechanistic insight into an observed kratom-drug interaction. Using mitragynine as a surrogate of the complex mixture, inhibition of intestinal and hepatic CYP3A (time-dependent and reversible) and hepatic CYP2D6 (reversible) by mitragynine was incorporated into the model based on *in vitro* observations.^17^ Following mitragynine administration, the PBPK model accurately reproduced the interaction with midazolam (AUCR, 1.28) *via* intestinal CYP3A inhibition and lack of interaction with dextromethorphan (AUCR, 1.08; Table 2). The simulated magnitude of the interaction (AUCR, 1.02) with midazolam was ~30% lower than observed (AUCR, 1.39) if only reversible CYP3A inhibition was considered, indicating that time-dependent inhibition contributes to the mechanism of the interaction. As has been reported with grapefruit juice, time-dependent inhibition of CYP3A can result in irreversible loss of protein, requiring *de novo* synthesis to restore enzyme activity over a time course dictated by the half-life of CYP3A protein.^32^ Chronic consumption of kratom could lead to a prolonged inhibitory effect, which would gradually reverse upon discontinuing kratom and regeneration of enzyme or enterocytes. The simulated interaction (AUCR, 1.28) was within ~10% of the observed interaction (AUCR, 1.39), indicating that the observed inhibition was mediated primarily by mitragynine and partly by other alkaloids shown to inhibit CYP3A *in vitro*, including paynantheine and speciogynine.^18^

**Figure 5:**
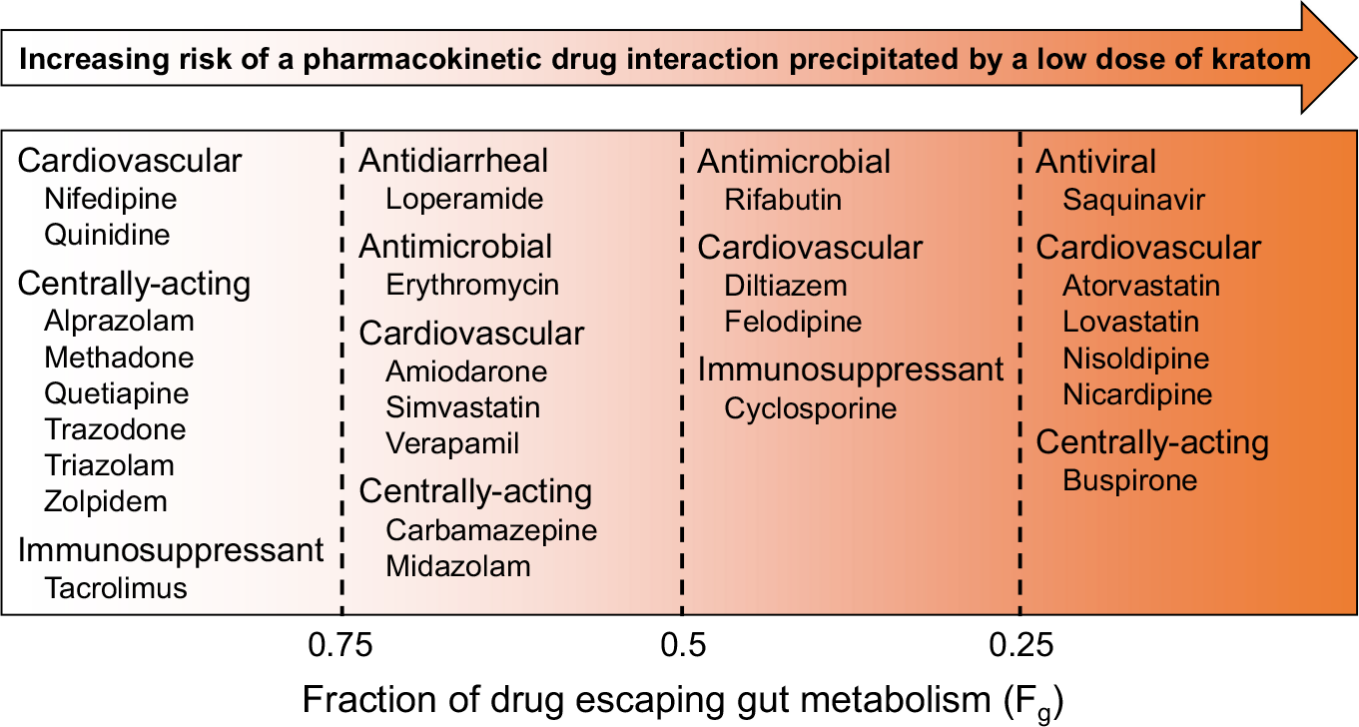
Examples of drugs that undergo intestinal CYP3A-mediated metabolism categorized based on the fraction of drug escaping intestinal metabolism (*F*_*g*_).^33–35^ Kratom was assumed not to alter fraction of the dose absorbed unchanged into enterocytes.

The extent of the observed pharmacokinetic kratom-midazolam interaction was modest with administration of a single low dose of kratom (2 g). That is, only orally administered drugs that undergo appreciable intestinal CYP3A-mediated first-pass metabolism^33–35^ will be affected (Figure 5). However, at higher doses and with chronic use, inhibition of hepatic CYP3A and CYP2D6 could result, widening the range of object drugs. Indeed, the dose consumed by habitual kratom users has been reported to be much higher than 2 g/day, up to 850 g/day.^36,37^ In addition, the concentration of mitragynine measured in postmortem autopsy blood was ~100-fold higher than that observed in the current study (Figure S3).^12^ These observations suggest that the decedents consumed a much higher dose of kratom, increasing the risk of a kratom-drug interaction. Our PBPK model could be applied to those scenarios.

There are limitations to this work. First, mitragynine is a time-dependent inhibitor of CYP3A *in vitro*,^17^ suggesting that the magnitude of interaction would be higher with chronic kratom consumption. Mitragynine has also been shown to be a weak inducer of CYP1A2 and CYP3A4.^38^ However, given that a single dose of mitragynine (~40 mg) was administered in this study, these time-dependent mechanisms could not be distinguished. Clinical studies involving chronic kratom or mitragynine administration are needed to determine whether a net inhibitory or inductive effect will result. Such a study is now feasible based on the safety data acquired from the present study. Second, given the subtherapeutic dose of midazolam, pharmacodynamic changes were not measured. Evaluating the effects of kratom on the pharmacodynamics of a therapeutic dose of midazolam or an alternate object drug is needed to assess the clinical significance of a pharmacokinetic kratom-drug interaction. Third, although less common, kratom has been reported to be administered *via* nonoral routes, including intravenous and inhalation.^39,40^ Our PBPK model could be refined to simulate potential drug interactions precipitated by kratom upon administration by nonoral routes.

In summary, the pharmacokinetic drug interaction potential of kratom was assessed *via* a proof-of-concept clinical study. Results suggest that a single low dose of kratom can modestly increase systemic exposure to co-consumed drugs that undergo extensive intestinal CYP3A-mediated first-pass metabolism. In contrast, the interaction risk is low for drugs eliminated predominately via CYP2D6-mediated metabolism (e.g., several selective serotonin reuptake inhibitors, beta blockers, and opioids). The verified PBPK models not only confirmed the mechanism of the CYP3A-mediated interaction but also can be used to predict potential pharmacokinetic interactions with other drugs commonly co-consumed with kratom. The effects of higher doses and chronic administration of kratom could also be simulated. The foundational clinical evidence from this study warrants additional research to enable regulatory agencies to make informed decisions about the safety of kratom when co-consumed with other drugs, thereby addressing ongoing public health concerns.

## Supplementary Material

Table S2

Table S1

Figure S3

Table S4

Table S3

Figure S1

Figure S2

## Figures and Tables

**Table 1:** Pharmacokinetics of the probe drugs and metabolites in the absence and presence of kratom tea administered to 12 healthy adult participants

Measure	Geometric mean (90% Confidence Interval)		
	Baseline	Kratom exposure	Kratom exposure/baseline
Midazolam			
AUC_inf_ (nM*h)^a^	58.3 (48.5–70.2)	80.8 (64.2–102)*	1.39 (1.23–1.57)
*C*_max_ (nM)	21.1 (17.2–26.0)	31.6 (26.4–38.0)*	1.50 (1.32–1.70)
*t*_max_ (h)	0.5 (0.5–1.0)^b^	0.5 (0.5–1.0)^b^	NA
*t*_1/2_ (h)	3.85 (3.25–4.56)	4.12 (3.45–4.92)	1.07 (0.98–1.17)
CL/*F* (L/h)	132 (110–159)	95.2 (75.7–120)*	0.72 (0.64–0.82)
*V_z_/F* (L)	732 (613–874)	566 (470–681)	0.77 (0.67–0.89)
CL_R_ (L/h)	0.06 (0.04–0.1)	0.05 (0.03–0.06)	0.77 (0.52–1.13)
1′-Hydroxymidazolam			
AUC_inf_ (nM*h)^a^	32.5 (27.2–38.8)	38.2 (31.7–46.1)	1.18 (1.05–1.32)
*C*_max_ (nM)	11.1 (9.30–13.3)	13.6 (12.2–15.2)	1.23 (1.03–1.46)
*t*_max_ (h)	0.75 (0.5–1.0)^b^	0.5 (0.5–1.0)^b^	NA
*t*_1/2_ (h)	4.28 (3.31–5.53)	5.81 (4.45–7.58)	1.35 (0.96–1.92)
Metabolic ratio^c^	0.56 (0.48–0.64)	0.48 (0.42–0.54)*	0.85 (0.77–0.93)
CL_R_ (L/h)	0.20 (0.15–0.27)	0.25 (0.17–0.37)	1.27 (0.81–2.0)
4-Hydroxymidazolam			
AUC_inf_ (nM*h)^a^	4.39 (3.74–5.15)	5.59 (4.75–6.59)*	1.27 (1.13–1.43)
*C*_max_ (nM)	1.28 (1.09–1.51)	1.81 (1.60–2.04)*	1.41 (1.25–1.58)
*t*_max_ (h)	1.0 (0.5–1.0)^b^	1.0 (0.5–1.0)^b^	NA
*t*_1/2_ (h)	3.57 (2.83–4.49)	3.24 (2.57–4.09)	0.91 (0.74–1.12)
Metabolic ratio^c^	0.076 (0.070–0.090)	0.070 (0.060–0.080)	0.92 (0.85–0.99)
CL_R_ (L/h)	0.15 (0.10–0.24)	0.09 (0.06–0.14)	0.59 (0.40–0.87)
Dextromethorphan			
AUC_inf_ (nM*h)^a^	46.5 (19.4–112)	46.2 (19.2–112)	0.99 (0.83–1.19)
*t*_max_ (h)	4.0 (2.0–6.0)^b^	4.0 (1.5–8.0)^b^	NA
*C*_max_ (nM)	4.57 (2.07–10.1)	4.3 (2.06–9.28)	0.96 (0.78–1.19)
*t*_1/2_ (h)	6.87 (5.85–8.06)	6.84 (5.62–8.34)	1.00 (0.92–1.08)
CL/*F* (L/h)	1740 (724–4,190)	1750 (727–4,230)	1.01 (0.84–1.21)
*V_z_/F* (L)	17,300 (8,200–36,400)	17,300 (8,330–36,000)	1.00 (0.82–1.23)
CL_R_ (L/h)	3.64 (2.41–5.51)	4.74 (3.41–6.60)	1.30 (0.88–1.93)
Dextrorphan			
AUC_inf_ (nM*h)^a^	68.4 (53.8–86.9)	74.0 (57.9–94.7)	1.08 (0.94–1.25)
*C*_max_ (nM)	10.9 (8.55–13.8)	11.5 (8.34–16.0)	1.06 (0.78–1.46)
*t*_max_ (h)	2.0 (1.5–6.0)^b^	3.0 (1.0–6.0)^b^	NA
*t*_1/2_ (h)	04684.25 (3.61–5.03)	4.57 (3.87–5.40)	1.07 (0.95–1.22)
Metabolic ratio^c^	1.47 (0.7–3.12)	1.60 (0.76–3.36)	1.09 (0.89–1.33)
CL_R_ (L/h)	16.3 (12.6–21.1)	17.2 (12.8–23.0)	1.05 (0.78–1.42)

Values represent geometric means (90% confidence interval).

AUC_inf_, area under the plasma concentration-time profiles from time zero to infinity; *C*_max_, maximum plasma concentration; CL/*F*, oral clearance; CL_R_, renal clearance; NA, not applicable; *t*_1/2_, terminal elimination half-life; *t*_max_, time to reach maximal concentration; *V_z_/F*, apparent volume of distribution during the terminal phase.

a The extrapolated AUC for midazolam, 1′-hydroxymidazolam, 4-hydroxymidazolam, and dextrorphan beyond the respective *C*_last_ was < 20%, and that for dextromethorphan was < 40%.

b Median (range).

c Calculated by dividing AUC_inf_ of metabolite by the AUC_inf_ of parent.

* *P* < 0.05 compared with baseline (Wilcoxon signed rank test).

**Table 2:** Comparison of clinical study outcomes to PBPK model simulations

Measure	Observed geometric mean (90% CI)	Simulated geometric mean (90% CI)
Midazolam (2.5 mg, oral)		
*t*_1/2_ (h)	3.85 (3.25–4.56)	4.35 (4.17–4.53)
*t*_max_ (h)	0.5 (0.5–1.0)^a^	1 (0.45–1.45)^a^
*C*_max_ (nM)	21.1 (17.2–26.0)	23.6 (21.5–26.0)
AUC_0–12h_ (nM*h)	53.7 (45.3–63.8)	78.5 (70.4–87.9)
AUC_inf_ (nM*h)	58.3 (48.5–70.2)	86.5 (76.6–97.7)
CL/*F* (L/h)	132 (110–159)	88.7 (78.5–100)
Treatment/baseline AUC_inf_ ratio	1.39 (1.23–1.57)	1.28 (1.26–1.30)
Treatment/baseline *C*_max_ ratio	1.50 (1.32–1.70)	1.24 (1.22–1.26)
Dextromethorphan (30 mg, oral)^b^		
*t*_1/2_ (h)	6.87 (5.85–8.06)	7.31 (6.91–7.74)
*t*_max_ (h)	4.0 (2.0–6.0)^a^	3.40 (2.15–4.70)^a^
*C*_max_ (nM)	4.57 (2.07–10.1)	4.93 (4.39–5.54)
AUC_0–12h_ (nM*h)	30.4 (13.6–67.9)	42.7 (38.1–47.9)
AUC_inf_ (nM*h)	46.5 (19.4–112)	68.8 (61.1–77.6)
CL/*F* (L/h)	2,380 (989–5,710)	1,180 (1050–1,330)
Treatment/baseline AUC_inf_ ratio	0.99 (0.83–1.19)	1.08 (1.07–1.09)
Treatment/baseline *C*_max_ ratio	0.96 (0.78–1.19)	1.08 (1.08–1.09)
Mitragynine (39 mg, oral)		
*t*_max_ (h)	1 (0.75–2)^a^	0.8 (0.55–1.15)^a^
*C*_max_ (nM)	119 (88–162)	106 (99.5–113)
AUC_0–24h_ (nM*h)	388 (282–533)	306 (284–328)

AUC_0–12h_, area under the concentration-time curve from zero to 12 hours; AUC_inf_, area under the concentration-time curve from time zero to infinity; CI, confidence interval; CL/*F*, oral clearance; *C*_max_, maximal concentration; PBPK, physiologically based pharmacokinetic; *t*_1/2_, terminal elimination half-life; *t*_max_, time to maximal concentration.

a Median (range).

b Equivalent to 22 mg free base.

## Study Highlights

### WHAT IS THE CURRENT KNOWLEDGE ON THE TOPIC?

- Kratom is a readily available botanical used to self-manage pain and opioid withdrawal symptoms. Overdose deaths associated with polyintoxication are increasingly reported. *In vitro-in vivo* extrapolation suggested a risk for pharmacokinetic kratom-drug interactions mediated *via* CYP3A but not CYP2D6 inhibition.

### WHAT QUESTION DID THIS STUDY ADDRESS?

- Does kratom alter the pharmacokinetics of drugs eliminated predominately *via* CYP3A- or CYP2D6-mediated metabolism in humans? Can a physiologically based pharmacokinetic (PBPK) model be developed to gain mechanistic insight into the observed effects?

### WHAT DOES THIS STUDY ADD TO OUR KNOWLEDGE?

- A single low dose of kratom precipitated a modest increase in the systemic exposure to midazolam (CYP3A probe) but not to that of dextromethorphan (CYP2D6 probe). The PBPK model suggested that time-dependent inhibition of intestinal CYP3A by mitragynine is the major underlying mechanism.

### HOW MIGHT THIS CHANGE CLINICAL PHARMACOLOGY OR TRANSLATIONAL SCIENCE?

- Clinical evidence of a pharmacokinetic kratom-drug interaction suggests that co-consuming kratom with other drugs extensively metabolized by intestinal CYP3A may precipitate serious interactions. This information can help guide policies for the safe and effective use of this emerging botanical.
