# Supplementary material for: Clinical Assessment of the Drug Interaction Potential of the Psychotropic Natural Product Kratom
Source: Clin Pharmacol Ther. Author manuscript; Available in PMC 2023 Jun 1. (PMC10198846; doi:10.1002/cpt.2891)
Supplement: Table S2 [file NIHMS1889761-supplement-Table_S2.docx]

**Table S2.** Bioanalytical method and procedures for plasma and urine

| **Analyte** | **Midazolam** | **1’-Hydroxy-midazolam** | **4-Hydroxy-midazolam** | **Dextromethorphan** | **Dextrorphan** |
| --- | --- | --- | --- | --- | --- |
| LC column | Reverse-phase, Acquity UPLC® HSS T3 column, 1.8 µm, 50 x 2.1 mm with a VanGuard™ pre-column at 40°C | | | | |
| Mobile phase | 0.1% formic acid in water (A) and 0.1% formic acid in acetonitrile (B) at 0.75 mL/min flow rate | | | | |
| Mobile phase gradient | 0-0.4 minutes, 10% B; 0.4-1.5 minutes, 10%-95% B; 1.5-2.0 minutes, 95% B; 2.0-2.1 minutes, 95%-10% B; 2.1-3.0 minutes, 10% B | | | | |
| Ionization mode | Positive | | | | |
| Internal standard | Midazolam-*d_4_* | | | Dextromethorphan-*d_3_* | |
| Transition (*m/z*) | 326.0 → 291.1 | 342.0 → 324.0 | 342.2 → 325.0 | 272.2 → 171.0 | 258.2 → 157.2 |
| Declustering potential (V) | 30 | 30 | 30 | 30 | 30 |
| Collision energy (V) | 40 | 30 | 33 | 40 | 47 |
| Retention time (min) | 1.28 | 1.26 | 1.22 | 1.26 | 1.13 |
