# Supplementary material for: Clinical Assessment of the Drug Interaction Potential of the Psychotropic Natural Product Kratom
Source: Clin Pharmacol Ther. Author manuscript; Available in PMC 2023 Jun 1. (PMC10198846; doi:10.1002/cpt.2891)
Supplement: Table S1 [file NIHMS1889761-supplement-Table_S1.docx]

**Table S1.** Clinical study inclusion and exclusion criteria.

| **Inclusion:**   - Aged from 18-55 years and healthy - Not taking any medications (prescription and non-prescription) or dietary/herbal supplements that can modulate the disposition of the study drugs and kratom alkaloids - Willing to abstain from consuming dietary/herbal supplements and citrus juices for several weeks - Willing to abstain from cannabis/marijuana, hemp, THC-, and CBD-containing products for several weeks - Willing to abstain from consuming caffeinated beverages or other caffeine-containing products the evening before each inpatient day - Willing to abstain from consuming any alcoholic beverages for one day prior to each inpatient day and outpatient visit - Willing to use an acceptable method of contraception that does not include oral contraceptive pills or patches (such as abstinence, copper IUD, condom) - Consumed kratom in the past without experiencing ill effects and are willing to abstain for at least two weeks prior to beginning the study |
| --- |
| **Exclusion:**   - Have never consumed kratom - Any current major illness or chronic illness such as (but not limited to) kidney disease, hepatic disease, diabetes mellitus, hypertension, coronary artery disease, chronic obstructive pulmonary disease, cancer, or HIV/AIDS - History of anemia or any other significant hematologic disorder - History of seizure disorder - History of drug or alcohol addiction or major psychiatric illness - Females who are pregnant or nursing - Need for chronic opioid pain medications - Have a history of intolerance or allergy to kratom, dextromethorphan/opioids, or midazolam/benzodiazepines - Used opioid pain medications within the last 3 weeks - Taking concomitant medications, both prescription and non-prescription (including dietary/herbal supplements and cannabis-derived products) that can modulate the disposition of the study drugs and kratom alkaloids |
