# Supplementary material for: Clinical Assessment of the Drug Interaction Potential of the Psychotropic Natural Product Kratom
Source: Clin Pharmacol Ther. Author manuscript; Available in PMC 2023 Jun 1. (PMC10198846; doi:10.1002/cpt.2891)

Figure S3. Plasma concentration vs. time profiles for the kratom alkaloids mitragynine, speciogynine, mitraciliatine, speciociliatine, paynantheine, isopaynantheine, and 7-hydroxymitragynine after a single low dose (2 g) of kratom tea administered 15 min prior to midazolam (2.5 mg) and dextromethorphan (30 mg) administration. Symbols and error bars denote geometric means and 90% confidence intervals, respectively, for 11 healthy adult participants.

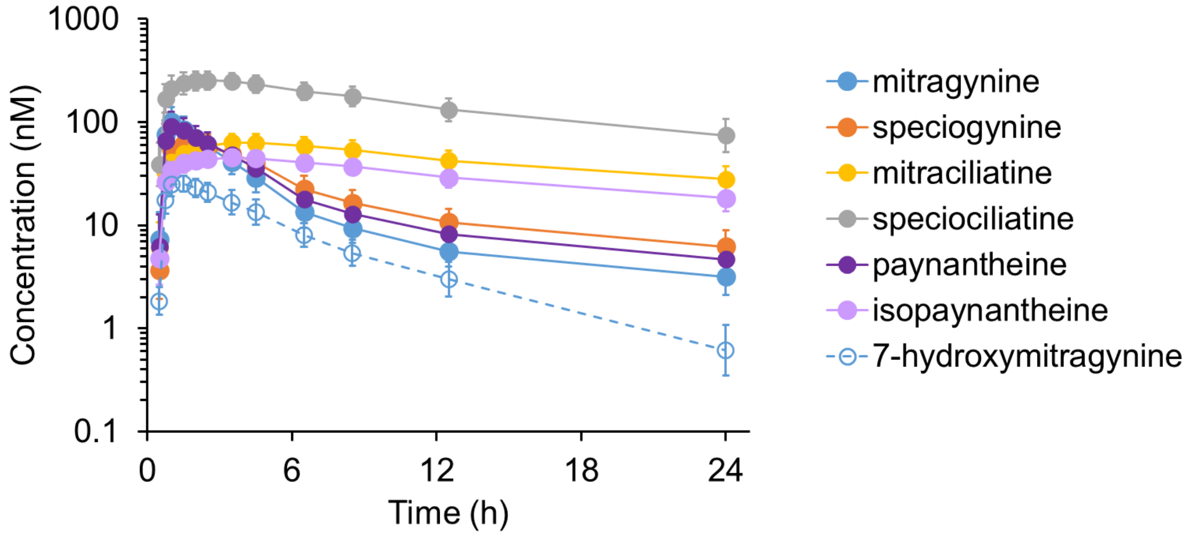

Supplement: Figure S3 [file NIHMS1889761-supplement-Figure_S3.pdf]
