# Supplementary material for: Clinical Assessment of the Drug Interaction Potential of the Psychotropic Natural Product Kratom
Source: Clin Pharmacol Ther. Author manuscript; Available in PMC 2023 Jun 1. (PMC10198846; doi:10.1002/cpt.2891)
Supplement: Table S4 [file NIHMS1889761-supplement-Table_S4.docx]

**Table S4.** Pharmacokinetics of kratom alkaloids in healthy adult participants (n=11) administered a kratom product (2 g) as a tea 15 min prior to midazolam (2.5 mg) and dextromethorphan (30 mg) administration.

| **Alkaloid** | **Measure** | **Geometric mean**  **(90% confidence interval)** |
| --- | --- | --- |
| mitragynine | t_max_ (h) | 1 (0.75-2)^a^ |
|  | C_max_ (nM) | 119 (88-162) |
|  | AUC_0-24h_ (nM*h) | 388 (282-533) |
| speciogynine | t_max_ (h) | 1.5 (0.75-3.5)^a^ |
|  | C_max_ (nM) | 79 (56-112) |
|  | AUC_0-24h_ (nM*h) | 465 (344-628) |
| mitraciliatine | t_max_ (h) | 3.5 (1-6.5)^a^ |
|  | C_max_ (nM) | 69 (55-85) |
|  | AUC_0-24h_ (nM*h) | 1054 (835-1331) |
| speciociliatine | t_max_ (h) | 2.5 (1-4.5)^a^ |
|  | C_max_ (nM) | 283 (223-360) |
|  | AUC_0-24h_ (nM*h) | 3570 (2760-4618) |
| paynantheine | t_max_ (h) | 1 (0.75-2)^a^ |
|  | C_max_ (nM) | 108 (77-151) |
|  | AUC_0-24h_ (nM*h) | 448 (333-602) |
| isopaynantheine | t_max_ (h) | 3.5 (1-4.5)^a^ |
|  | C_max_ (nM) | 50 (41-62) |
|  | AUC_0-24h_ (nM*h) | 732 (584-918) |
| 7-hydroxymitragynine | t_max_ (h) | 1 (0.75-2.5)^a^ |
|  | C_max_ (nM) | 31 (24-40) |
|  | AUC_0-24h_ (nM*h) | 151 (114-200) |

^a^median (range). C_max_, maximum plasma concentration; t_max_, time to reach C_max_; AUC_0-24h_, area under the plasma-concentration time curve from time zero to 24 hours.
