# Supplementary material for: Clinical Assessment of the Drug Interaction Potential of the Psychotropic Natural Product Kratom
Source: Clin Pharmacol Ther. Author manuscript; Available in PMC 2023 Jun 1. (PMC10198846; doi:10.1002/cpt.2891)
Supplement: Table S3 [file NIHMS1889761-supplement-Table_S3.docx]

**Table S3.** Simcyp model input parameters for mitragynine (surrogate alkaloid for kratom), midazolam, and dextromethorphan to simulate the clinical kratom-drug interaction study.

| **Input** | **Mitragynine** | **Midazolam HCl^a^** | **Dextromethorphan HBr^b^** |
| --- | --- | --- | --- |
| Dose (mg) | 39^c^ | 2.5 | 30 (22)^d^ |
| Physicochemical and blood binding properties | | | |
| MW (g/mol) | 398.5 | 325.8 | 271.4 |
| Compound type | Monoprotic Base^e^ | Ampholyte | Monoprotic base |
| pK_a1_ | 7.68^e^ | 10.95 | 8.3 |
| pK_a2_ | - | 6.2 | - |
| LogP | 4.8^f^ | 3.53 | 3.8 |
| B/P | 0.93^g^ | 0.603 | 1.32 |
| f_u,p_ | 0.04^g^ | 0.032 | 0.5 |
| Absorption | | | |
| Absorption model | ADAM | 1^st^ order | 1^st^ order |
| f_a_ | - | 1 | 1 |
| k_a_ (h^-1^) | - | 1.42 | 0.6 |
| f_u,gut_ | 1 | 1 | 1 |
| Q_gut_ (L/h) | - | 16.18 | 19.03 |
| P_eff_ (10^-4^ cm/s) | 2.98 | 6.05 | 12 |
| P_app_ (10^-6^ cm/s) | - | 213 | - |
| PSA (Å^2^) | 63.8^e^ | - | 12.5 |
| HBD | 1^e^ | - | 0 |
| Distribution | | | |
| Distribution model | Full PBPK | Minimal PBPK | Minimal PBPK |
| V_ss_ (L/kg) | 46.5 (predicted, method 2) | 0.88 | 14.5 |
| k_in_ (h^-1^) | - | 0.2 | - |
| k_out_ (h^-1^) | - | 0.25 | - |
| Q (L/h) | - | - | 15 |
| V_sac_ (L/kg) | - | 0.23 | 1 |
| Elimination | | | |
| Clearance type | In vivo clearance | Enzyme kinetics | Enzyme kinetics |
| CL_po_ (L/h) | 233 (52% CV)^g^ | - | - |
| Enzyme kinetics  (μM, K_m_;  pmol/min/pmol of isoform, V_max_;  μL/min/mg of protein, CL_int_) | - | rCYP3A4_met1_  2.16 - K_m_  5.23 - V_max_ | CL_int_ (met1)  CYP2B6 - 0.03  CYP2C9 - 0.76  CYP2C18 - 0.01 CYP2C19 - 0.05  CYP2D6 - 678  CYP3A4 - 1.13 |
|  |  | rCYP3A5_met1_  4.16 - K_m_  19.7 - V_max_ |  |
|  |  | rCYP3A4_met2_  31.8 - K_m_  5.2 - V_max_ |  |
|  |  | rCYP3A5_met2_  38.4 - K_m_  4.03 - V_max_ | CL_int_ (met2)  CYP2B6 - 4.74  CYP2C9 - 0.14  CYP2C18 - 0.05 CYP2C19 - 0.07  CYP2D6 - 6.08  CYP3A4 - 3.02 |
| CL_R_ (L/h) | 0.19^g^ | 0.085 | 0.375 |
| Interaction | | | |
| Enzyme | CYP2D6  Reversible inhibition^h^  K_i_ - 1.17 μM  f_u,mic_ - 0.95^i^ | - | - |
|  | CYP3A  Reversible inhibition^h^  K_i_ - 10.2 μM  f_u,mic_ - 0.95^i^ | - | - |
|  | CYP3A  Time-dependent inhibition^h^  K_I_ - 1.17 μM  k_inact_ - 4.5 h^-1^  f_u,mic_ - 0.95^i^ | - | - |

MW, molecular weight; f_u,mic_, fraction unbound in microsomal incubation; K_i_, reversible inhibition constant; K_I_, time-dependent inhibition constant; k_inact_, maximum rate of inactivation; HIMs, human intestinal microsomes; HLMs, human liver microsomes; CL_R_, renal clearance; K_m_, Michaelis-Menten constant; V_max_, maximum velocity; CL/F, oral clearance; %CV, % coefficient of variation; V_ss_, steady-state volume of distribution; V_sac_, volume of the single adjusting compartment; k_in_ and k_out_, first-order rate constants for compounds into and out of the single adjusting compartment (SAC); Q, distribution clearance to the SAC; HBD, hydrogen bond donor; PSA, polar surface area; pK_a1_ and pK_a2_, log ionization constants; LogP, lipophilicity; B/P, blood to plasma ratio; f_u,p_, fraction unbound in plasma; f_a_, fraction absorbed; k_a_, absorption rate constant; f_u,gut_, fraction unbound in gut; Q_gut_, model to estimate gut first pass metabolism; P_eff_, effective permeability; P_app_, apparent permeability. Modified ^a^midazolam and ^b^dextromethorphan models based on literature;^21,22^ ^c^2 g of kratom product contains 19.48x2 mg mitragynine; ^d^equivalent to 22 mg free base; ^e^obtained using ADMET predictor; ^f^logP estimated to fit the observed plasma concentration-time curve; ^g^mitragynine pharmacokinetics and blood binding parameters were previously determined;^19^ ^h^inhibition mechanism and parameters were previously determined; the K_i_ for CYP3A was calculated from the IC_50_ using the Cheng-Prusoff equation assuming competitive inhibition (K_i_ = IC_50_/2) ;^17^ ^i^f_u,mic_ previously determined using 0.05 mg/mL of human liver microsomes matching the CYP2D6 and CYP3A inhibition experiments.

**Mitragynine PBPK model development.** The mitragynine PBPK model was developed using a middle-out technique. Available physicochemical parameters for mitragynine were obtained from the literature or PubChem or were predicted using ADMET Predictor (v10.4; Simulations Plus Inc., Lancaster, CA). Plasma and blood binding parameters were determined previously.18 Mitragynine absorption after oral administration of kratom tea was described using the Advanced Dissolution, Absorption and Metabolism (ADAM) model within Simcyp. Volume of distribution at steady state (V_ss_) and tissue:plasma partition coefficients were calculated using the Rodgers and Rowland method (method 2). Elimination was defined using oral clearance obtained from our previous clinical kratom pharmacokinetic study in which a single low dose (2 g) of the K51 product was administered to six healthy participants.^1^ Mitragynine is mainly eliminated by metabolism with minimal renal excretion as defined by a low renal clearance.^1^ Mitragynine model development was guided by the plasma concentration-time profiles observed in our previous study to capture the biphasic profile.

**Reference**

1. Tanna, R.S. et al. Clinical pharmacokinetic assessment of kratom (*Mitragyna speciosa*), a botanical product with opioid-like effects, in healthy adult participants. *Pharmaceutics* **14**, 620 (2022).
