# Supplementary material for: Clinical Assessment of the Drug Interaction Potential of the Psychotropic Natural Product Kratom
Source: Clin Pharmacol Ther. Author manuscript; Available in PMC 2023 Jun 1. (PMC10198846; doi:10.1002/cpt.2891)

Figure S1. Comparison of heart rate (a), blood oxygen saturation (b), and blood pressure (c) for 12 healthy adult participants after administration of single doses of the probe drugs midazolam (2.5 mg) and dextromethorphan (30 mg) alone (baseline) and after administration of a single low dose (2 g) of kratom tea (kratom exposure). Symbols and error bars denote means and standard deviations, respectively. Sys., systolic; Dia., diastolic.

(a) Heart rate

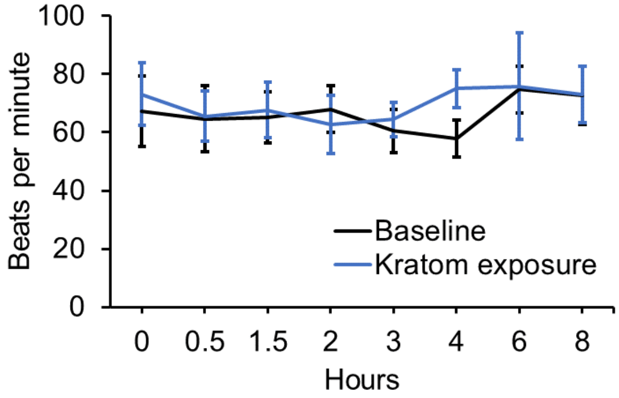

(b) Blood oxygen saturation

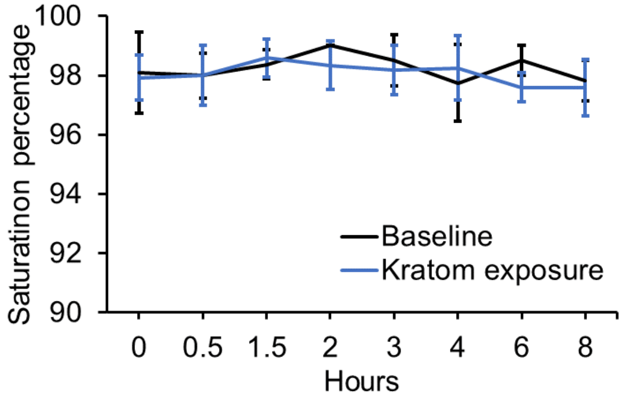

(c) Blood pressure

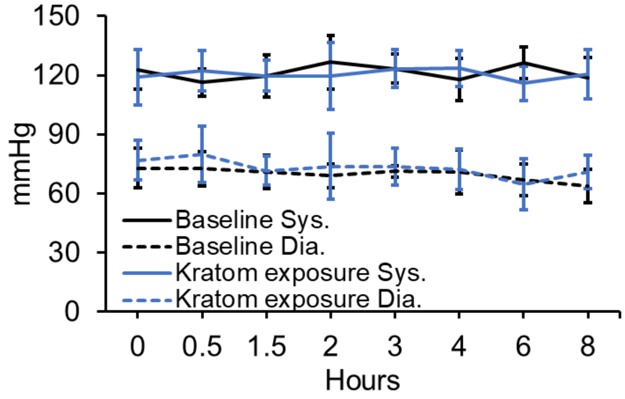

Supplement: Figure S1 [file NIHMS1889761-supplement-Figure_S1.pdf]
