# Supplementary material for: Clinical Assessment of the Drug Interaction Potential of the Psychotropic Natural Product Kratom
Source: Clin Pharmacol Ther. Author manuscript; Available in PMC 2023 Jun 1. (PMC10198846; doi:10.1002/cpt.2891)

Figure S2. Simulated percentage of active (a) CYP3A4 and (b) CYP3A5 remaining in small intestine, colon, and liver after time-dependent inhibition of the enzyme activity *via* a single low mitragynine dose (38.96 mg).

(a) CYP3A4 (b) CYP3A5

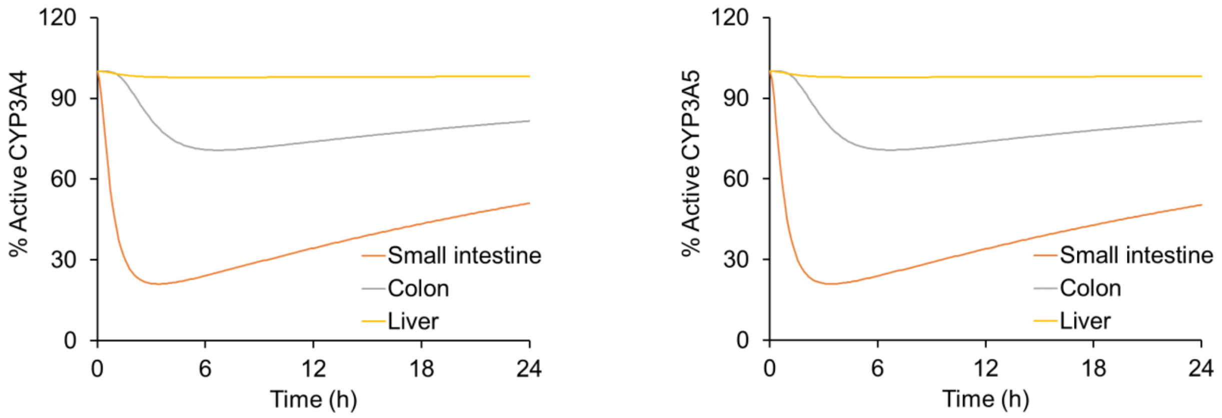

Supplement: Figure S2 [file NIHMS1889761-supplement-Figure_S2.pdf]
